# Electrical activity of the diaphragm (EAdi) as a monitoring parameter in difficult weaning from respirator: a pilot study

**DOI:** 10.1186/cc12865

**Published:** 2013-08-28

**Authors:** Jürgen Barwing, Cristina Pedroni, Ulrike Olgemöller, Michael Quintel, Onnen Moerer

**Affiliations:** 1 Department of Anesthesiology, Emergency and Intensive Care Medicine, University of Göttingen Medical School (Georg-August University of Goettingen), Robert-Koch-Str. 40, D-37099 Göttingen, Germany; 2 Scuola di Specializzazione in Anestesia, Rianimazione e Terapia Antalgica, Università degli Studi di Pavia, IRCCS Policlinico S.Matteo, Viale Camillo Golgi, 19, 27100 Pavia, Province of Pavia, Italy; 3 Dipartimento di Anestesia e Rianimazione 1°, Ospedale S. Bortolo di Vicenza, viale Rodolfi, 37-36100 Vicenza, Italy

**Keywords:** Electrical activity of the diaphragm (EAdi), Neurally adjusted ventilatory assist (NAVA), Ventilator weaning

## Abstract

**Introduction:**

A reliable prediction of successful weaning from respiratory support may be crucial for the overall outcome of the critically ill patient. The electrical activity of the diaphragm (EAdi) allows one to monitor the patients’ respiratory drive and their ability to meet the increased respiratory demand. In this pilot study, we compared the EAdi with conventional parameters of weaning failure, such as the ratio of respiratory rate to tidal volume.

**Methods:**

We studied 18 mechanically ventilated patients considered difficult to wean. For a spontaneous breathing trial (SBT), the patients were disconnected from the ventilator and given oxygen through a T-piece. The SBT was evaluated by using standard criteria.

**Results:**

Twelve patients completed the SBT successfully, and six failed. The EAdi was significantly different in the two groups. We found an early increase in EAdi in the failing patients that was more pronounced than in any of the patients who successfully passed the SBT. Changes in EAdi predicted an SBT failure earlier than did conventional parameters.

**Conclusions:**

EAdi monitoring adds valuable information during weaning from the ventilator and may help to identify patients who are not ready for discontinuation of respiratory support.

## Introduction

Successful weaning from respiratory support is a matter of major importance in critically ill patients recovering from acute respiratory failure. The assessment of lung function before discontinuation of ventilator support is essential to predict the patient’s ability to breathe spontaneously without mechanical assistance. Weaning readiness has been the subject of research over the past years, and numerous parameters have been proposed as predictors of weaning success, indicating the lack of a reliable solution. An easy-to-obtain and therefore widely accepted parameter is the rapid shallow breathing index (RSBI), which is the ratio of respiratory rate and tidal volume (RR/VT) [1,2].

Electromyography of the diaphragm has been used to describe the patients’ functional status and neuromuscular coupling in numerous studies [3-5] and has been shown to provide a real-time, breath-by-breath measure of neural respiratory drive [6]. With the clinical introduction of neurally adjusted ventilatory assist (NAVA) as a ventilator mode, the electrical activity of the diaphragm (EAdi) gives the clinician a monitoring parameter of diaphragmatic function. The electromyogram of the crural diaphragm is recorded by a set of electrodes incorporated into a special nasogastric tube (EAdi catheter). The filtered and amplified signal is displayed on the ventilator’s control panel [7]. Experimental studies have shown its value in measuring diaphragmatic function [8,9], as the amplitude of the signal correlates with the patients’ respiratory drive [3,10]. Clinical studies and results from animal experiments confirmed the physiological concepts [11,12]. Although primarily intended to control the ventilator, the EAdi allows monitoring of the respiratory drive in relation to tidal volume. The tidal volume (VT) during unassisted spontaneous breathing is proportional to the electrical activity of the diaphragm and reflects the neural output of the central respiratory regulation in healthy dogs [8].

The diaphragmatic electromyogram has been used to describe respiratory effort in patients [13]. In patients with poor neuromuscular coupling, tidal-volume generation might not follow the patients’ central respiratory drive. As shown by Sinderby and colleagues [14], severe chronic obstructive pulmonary disease and restrictive pulmonary disease induce increased diaphragm activation at rest. During weaning, respiratory drive and thus EAdi are expected to increase earlier than conventional assessment parameters of muscular function (for example, tidal volume, respiratory rate, gas exchange, and clinical signs of exhaustion). The EAdi signal might therefore be a better monitoring parameter during weaning. Recently it was shown that EAdi-derived indices might provide reliable, early predictors of weaning outcome [15], and EAdi has also been used to monitor weaning from the ventilator in pediatric patients [16]

In this pilot study, we compared the EAdi with conventional weaning parameters in difficult-to-wean patients scheduled for an SBT. Our primary goal was to observe the expected increase in the EAdi. A secondary goal was to compare the conventional weaning parameters with those derived from the EAdi.

## Materials and methods

The study was performed in the anesthesiologic intensive care unit (ICU) at the University Hospital in Göttingen, Germany, after approval by the local ethics committee (Ethic Committee of the Medical Faculty, Georg-August-University, Göttingen). Written informed consent was waived because of the observational design of the study. All patients were recovering from respiratory failure after severe illness and were considered difficult to wean. These patients had either already failed an initial weaning attempt or had been on the ventilator for a long period and were expected to require more than 7 days for successful weaning. Patients who proceeded from initiation of weaning to extubation on the first attempt were excluded [17].

We identified patients as ready to wean by using criteria adapted from Macintyre *et al*. [18]:

– Age >18 years, capable of assisted spontaneous breathing

– RSBI <105

– Stable hemodynamics (heart rate (HR) ≤110 beats per minute (bpm), low-dose catecholamine therapy (epinephrine/norepinephrine ≤0.1 μg/kg/min)

– Positive end-expiratory pressure (PEEP) ≥8 cm H_2_O, FiO_2_ ≤0.5

– No acidosis (pH ≥7,35)

– Afebrile (temperature <38°C)

– Cooperative patients with motor-activity assessment scale (MAAS) scores from 2 to 4 [19]

All patients had a stable respiratory pattern at least 1 hour before starting the SBT. The patients were disconnected from the ventilator, and supplementary oxygen was supplied through a T-piece to keep peripheral oxygen saturation >90%.

The SBT was discontinued after 30 minutes or when one of the following events occurred [18]:

– Oxygen saturation <90%

– HR >120 bpm or change of HR >20%

– Systolic blood pressure (BP) >180 mm Hg or <90 mm Hg or change of BP >20%

– Respiratory rate (RR) ≥35 breath/minute or change of RR >50%

– Clinical signs of distress (change in mental status (MAAS 6), diaphoresis)

The correct position of the EAdi catheter was verified before the trial as described elsewhere [20]. The VT/EAdi ratio was calculated according to Wolf [16] by using VT (ml) and delta EAdi (EAdi_max_ - EAdi_min_) to quantify the patients’ electrical effort with tidal volume. We used respiratory-inductive plethysmography (RIP) with a thoracic band at mammillary level and an abdominal band at the level of the naval. Baseline measurements were used to calibrate tidal volume measured by RIP with flow-measured values from the ventilator by using a simplified qualitative diagnostic calibration (QDC) [21], assuming an equal contribution of thoracic and abdominal expansion to VT. RIP data were then used to estimate tidal volume during the disconnection period in the first 11 patients (VT_est_). For patients 12 to 18, we used an additional flow sensor (Bicore II; CareFusion, San Diego, CA, USA) to measure tidal volume. Relative changes in lung volume were derived for each patient by using RIP [22,23].

### Data collection

Data were recorded 5 minutes before the patient was disconnected from the ventilator (pre D), 5 minutes after disconnection (D (f)), before reconnection to the ventilator (D (l)) and 5 minutes after reconnection (post D). All RIP and EAdi-catheter data were recorded at a sampling rate of 60 to 100 Hz by using dedicated software (Bicore IIcker; Maquet, Solna, Sweden). Tidal volume, respiratory rate, PEEP, mean and peak airway pressure, EAdi_max_ and EAdi_min_, were taken from the NAVA tracker, analyzed breath-by-breath during a 2-minute measurement period, and the mean values were used for further analysis. Oxygen saturation, hemodynamic data (arterial blood pressure and heart rate), and arterial blood gases were collected at each step by a patient data-management system (ICIP; Philips, Eindhoven, The Netherlands). The mental status of the patient was assessed by the study group at each step by using the motor-activity assessment scale [19]. Outcome data were collected 72 hours after the trial (72 h) and at the end of the ICU stay.

### Statistical analysis

Because of the pilot character of the study, no sample-size calculation was performed. Descriptive data are given as medians and 25% and 75% quartiles, unless stated otherwise. Groups were tested for significant differences with the Mann–Whitney *U* test; paired variables were tested for significant differences with the Wilcoxon test, and multiple variables with the Friedman-ANOVA by using standard statistical software (Statistica 9.0; StatSoft, Tulsa, OK,USA).

## Results

Table 1 depicts basic patient data of the 18 patients studied. Twelve patients successfully completed the SBT (success patients), and six failed (failure patients). Based on the failure criteria defined in the Methods section [18], in 16 instances, failure criteria were met. A single patient could have more than one failed criterion. Reasons for failure were the following: an oxygen saturation >90% in two patients, an HR >120 bpm or a >20% change in HR in two patients, systolic BP >180 mm Hg or a >20% change in BP in four patients, a RR >35 breath/minute or a >50% change in RR in three patients (both criteria occurred in two patients), or clinical signs of distress in three patients.

**Table 1:** Basic patient data

**Number**	**F/S**	**Primary diagnosis**	**BMI**	**SAPS II a**	**SAPS II i**	**ICU d**	**Mode**	**PS (cm H**_**2**_**O)**	**PEEP (cm H**_**2**_**O)**	**EAdi**_**max **_**(μV)**	**FiO**_**2 **_**(%)**	**SpO**_**2 **_**(%)**	**D (min)**
1.	F	Sepsis	39	63	58	29	PSV	7	8	8	35	99	20
2.	F	ACS (CAB)	22	23	44	4	PSV	11	8	1	45	99	5
3.	S	AS (AVR)	27	39	37	13	PSV	6	5	10	40	100	30
4.	S	AS (AVR)	23	60	41	8	PSV	9	5	7	40	98	30
5.	F	Thorax apertum (CAB)	22	35	39	7	PSV	9	5	7	45	96	5
6.	S	AAA	24	54	46	5	PSV	7	6	5	35	97	30
7.	F	AS (AVR)	27	41	57	31	NAVA	(18)	6	9	50	98	30
8.	F	Spinal trauma C5-6	21	24	19	4	NAVA	(12)	5	12	45	99	15
9.	F	MI (MVR)	34	47	42	14	PSV	8	8	3	40	100	22
10.	S	ACS (CAB)	21	39	45	15	PSV	6	5	3	50	99	30
11.	S	SCLC	27	37	35	9	PSV	10	8	5	45	100	30
12.	S	ARDS	29	66	33	26	PSV	8	5	12	40	98	30
13.	S	Thorax trauma	29	51	35	22	PSV	6	6	58	30	96	30
14.	S	ACS (CAB)	24	40	46	10	PSV	10	7	10	35	99	30
15.	S	TI (TVR)	33	44	38	3	PSV	11	5	13	30	97	30
16.	S	ARDS	30	25	14	24	PSV	12	8	35	35	99	30
17.	S	Sepsis	22	46	28	46	NAVA	(16)	9	47	30	93	30
18.	S	Sepsis	43	42	51	10	NAVA	(14)	8	10	35	98	30
**Mean**			**28**	**43**	**39**	**16**		**10**	**7**	**14**	**39**	**98**	**25**
**STD**			**6**	**13**	**11**	**12**		**3**	**1**	**16**	**6**	**2**	**9**

*F/S* failure (F) or success (S), *BMI* body mass index, *SAPS II (a)* simplified acute physiology score II (on ICU admission), *SAPS II (i)* simplified acute physiology score II (day of inclusion in trial), *ICU (d)* day of ICU stay on study inclusion, *mode* ventilator mode, *PS (cm H*_2_*O)* pressure support in cm H_2_O, calculated maximum pressure support in NAVA mode in brackets, *PEEP (cm H*_2_*O)* positive end-expiratory pressure in cm H_2_O, *EAdi*_*max*_* (μV)* maximum electrical activity of the diaphragm during inspiration in microvolts, *FiO*_2_*(%)* fraction of inspiratory oxygen in percentage, *SpO*_2_*(%)* pulse oximetry oxygen saturation in percentage, *D (min)* time of disconnection from the ventilator during spontaneous-breathing trial, *M* male, *F* female, *ACS (CAB)* acute coronary syndrome treated with coronary artery bypass; AS (AVR), aortic valve stenosis treated with aortic valve replacement; AAA (AR), abdominal aortic aneurysm treated with aortic replacement, *CST* cervical spinal trauma, *MI (MVR)* mitral valve insufficiency treated with mitral valve replacement, *SCLC* small cell lung cancer, *STD* standard deviation (mean values and standard deviations of the numeric data are displayed at the bottom line). Calculated maximum pressure support in NAVA = EAdi_max_ (μV) ×NAVA level (cm H_2_O/μV).

**Figure 1:**
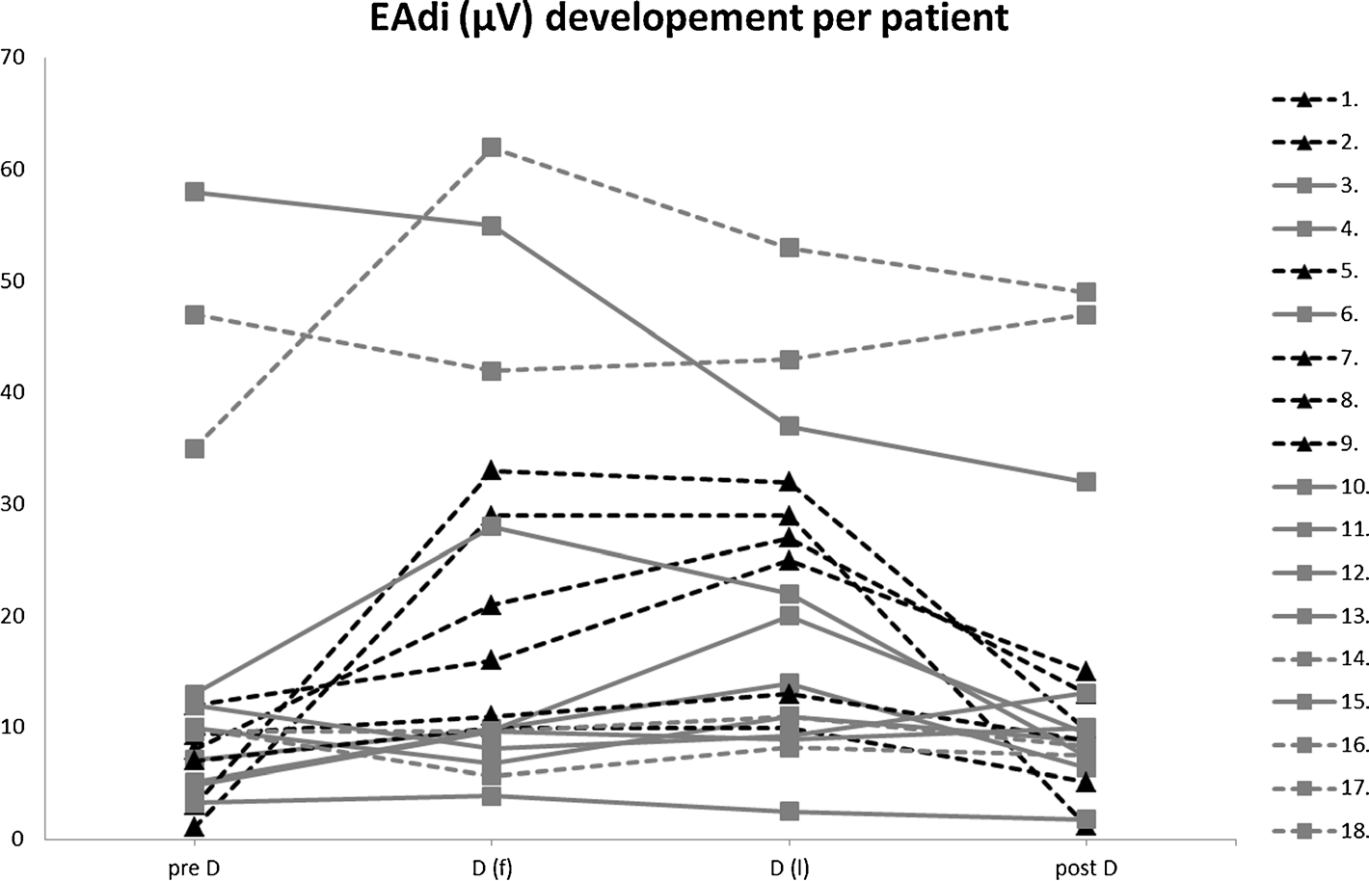
**EAdi**_**max **_**values of patients before, during, and after the spontaneous breathing trial.** The figure shows the EAdi_max_ values in microvolts for each patient before (pre D) during (D(f) and D(l)), and after a spontaneous breathing trial (SBT). Light grey squares depict EAdi_max_ values of patients successfully passing the SBT (*n* = 12), with dashed lines highlighting those patients still needing ventilator support after 72 hours (*n* = 4 of 12). Black triangles depict EAdi_max_ values of patients failing the SBT (*n* = 6). pre D, baseline values before SBT; D(f), first values after disconnection from the ventilator (after about 5 minutes); D(l), last values before reconnecting to the ventilator; post D, values after about 5 minutes after reconnecting to the ventilator.

With regard to the outcome after 72 hours, none of the six patients who failed the SBT was able to be weaned from mechanical ventilation, whereas eight of the 12 patients who passed the SBT were successfully weaned. Of the remaining four patients, three were already given a tracheotomy before the SBT and were classified as “prolonged weaning” [17]. The remaining patient successfully passing the SBT, who could not be weaned from the ventilator, received a tracheostomy later. At study inclusion, 10 patients had a tracheostomy; three of these failed the SBT. In the failure group, three of the six patients died in the ICU. This is consistent with the high SAPS II scores of our patients. No significant difference was found in gender, BMI, SAPS II, duration of ICU stay, duration of ventilator support, PEEP, and pressure support before study inclusion between failure and success patients. Disconnection from the ventilator led to changes in the weaning indices, EAdi_max_ (Figure 1), heart rate, and blood pressure in all patients (Table 2).

**Table 2:** Respiratory and hemodynamic parameters during the baseline measurements (pre D), the spontaneous breathing trial (D(f)) and (D(l)) and after reconnection to the ventilator (post D) for all patients

**Parameter**	**Time**	**All (*n *** **= 18)**	**Failure (*n *** **= 6)**	**Success (*n *** **= 12)**
VT (ml)	pre D	383 (339/508)^c^	340 (308/378)	423 (373/534)
	D (f)	337 (266/551)^c^	210 (167/470)	345 (297/475)
	D (l)	318 (220/453)^c^	198 (176/395)	343 (300/452)
	post D	409 (361/496)^c^	383 (344/462)	407 (362/514)
RR (bpm)	pre D	21 (16/29)	25 (20/30)	22 (15/26)
	D (f)	24 (20/29)	30 (25/38)^a^	21 (17/24)^a^
	D (l)	22 (20/30)	29 (23/37)^a^	21 (17/22)^a^
	post D	21 (17/24)	19 (17/21)	22 (18/23)
VT/EAdi	pre D	46 (29/120)^d^	45 (38/127)^c^	43 (25/92)
	D (f)	21 (11/43)^d^	14 (12/18)^c^	35 (15/70)
	D (l)	16 (8/27)^d^	14 (9/15)^c^	24 (12/53)
	post D	45 (27/68)^d^	41 (36/62)^c^	45 (23/64)
RR/VT	pre D	49 (37/81)^c^	77 (54/96)	48 (33/58)
	D (f)	61 (47/109)^c^	127 (62/286)	59 (44/74)
	D (l)	71 (51/118)^c^	135 (74/250)	63 (40/75)
	Post D	50 (33/71)^c^	51 (37/57)	50 (40/67)
HF (bpm)	Pre D	84 (79/91)^b^	84 (81/89)^b^	84 (76/88)
	D (f)	89 (79/102)^b^	87 (79/100)^b^	85 (80/95)
	D (l)	92 (78/103)^b^	100 (86/111)^b^	89 (77/95)
	Post D	88 (78/100)^b^	86 (79/97)^b^	89 (78/94)
BP syst (mm Hg)	Pre D	126 (111/142)^b^	125 (111/151)^c^	126 (118/139)
	D (f)	144 (121/151)^b^	162 (145/171)^c^	138 (116/147)
	D (l)	134 (121/150)^b^	155 (137/171)^c^	125 (121/145)
	post D	127 (116/140)^b^	133 (117/140)^c^	126 (121/133)
MAAS	pre D	3 (2/3)^d^	2 (2/3)^b^	3 (2/3)
	D (f)	3 (2/4)^d^	3 (2/4)^b^	3 (3/3)
	D (l)	3 (3/5)^d^	5 (3/5)^b^	3 (3/3)
	post D	3 (2/3)^d^	2 (2/3)^b^	3 (2/3)
EAdi_max_ (μV)	pre D	9 (5/12)^c^	8 (4/9)^c^	10 (7/19)
	D (f)	11 (10/29)^c^	19 (12/27)^c^	10 (8/32)
	D (l)	18 (11/29)^c^	26 (16/29)^c^	13 (9/26)
	post D	10 (8/15)^c^	9 (6/12)^c†^	9 (7/18)

During disconnection, weaning indices and clinical parameters changed significantly over all patients. ^a^*P* < 0.05 (tested: failure versus success; Mann–Whitney *U* test). ^b^*P* < 0.05 (tested: all, failure, success; Friedman ANOVA); ^c^*P* < 0.01 (tested: all, failure, success; Friedman ANOVA); ^d^*P* < 0.001 (tested: all, failure, success; Friedman ANOVA); data displayed as median with 25%/75% quartiles; pre D, baseline values before spontaneous breathing trial (SBT); D(f), first values after disconnection from the ventilator (after about 5minutes); D(l), last values before reconnecting to the ventilator; post D, values about 5 minutes after reconnecting to the ventilator; VT (ml), tidal volume in milliliter, *values estimated by respiratory inductive plethysmography (RIP) printed in italics*; RR (bpm), respiratory rate in beats per minute; VT/EAdi, tidal volume/EAdi_max_ quotient in milliliters/μV; RR/VT (L), respiratory rate/tidal volume quotient in beats per minute/liter; HF, heart frequency in beats per minute; BP syst, systolic blood pressure in mm Hg; MAAS, Motor Activity Assessment Scale.

**Figure 2:**
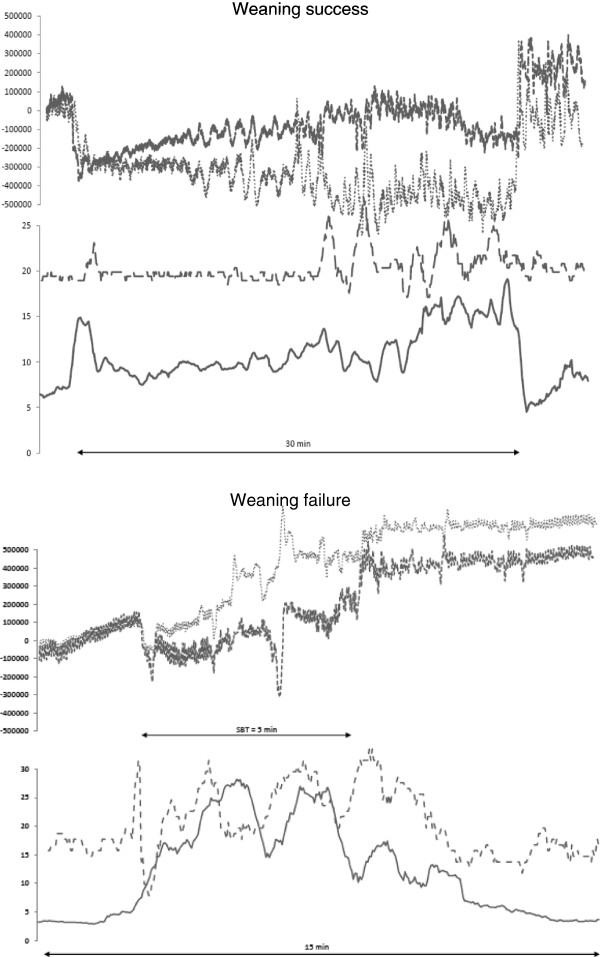
**Electrical activity of the diaphragm (EAdi**_**max**_**), respiratory inductive plethysmography (RIP), and respiratory rate (RR) tracings (y-axes) over time (x-axis) of a success (number 6) and a failure patient (number 2) during a spontaneous breathing trial (SBT).** The figure shows patient 6 successfully passing the SBT (top), and patient 2, who failed to pass the SBT (bottom). The horizontal line (number 6 SBT, 30 minutes; number 2 SBT, 5 minutes) indicates the duration of the SBT. In each patient, the upper part of the graph shows the RIP tracings from the thoracic (fine dots) and abdominal band (bold dots); the lower part shows the respiratory rate (dashed line) and the EAdi_max_ (solid line). Note the steep increase in EAdi_max_ in patient 2 after disconnection from the ventilator.

**Figure 3:**
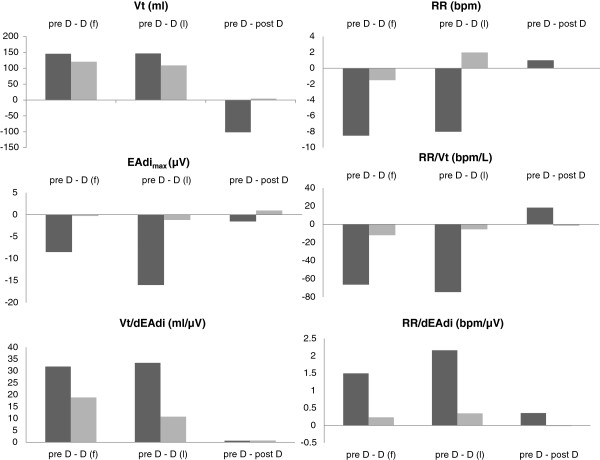
**Changes of tidal volume, respiratory rate, electrical activity of the diaphragm, and weaning indices in relation to pre D in failure (*n *= 6) and success (*n *= 12) patients.** The figure shows the median differences between D(f) and pre D, D(l) and pre D, and post D and pre D for tidal volume (VT), electrical activity of the diaphragm (EAdi_max_), respiratory rate (RR), and derived indices (RR/dEAdi, VT/dEAdi, and RR/VT) for failure and success patients. Dark columns show failure patients; light columns, success patients.

Respiratory rate during the SBT differed significantly between the failure and success groups (*P* < 0.05). Large differences occurred in other parameters (VT, VT/EAdi, RR/VT, BP, EAdi_max_), but these did not reach a statistically significant level (Table 2). During the SBT, significant changes were noted in VT/EAdi, HF, BP, MAAS, and EAdi_max_ in the group of failure patients, which were not observed in the success group (Table 2). These results are illustrated by the tracings of two patients in Figure 2. The EAdi_max_ tracing of the success patient shows a stable course, increasing to a moderate degree only late during the disconnection, whereas the EAdi of the failure patient increased immediately after disconnection from the ventilator. The changes during the SBT in relation to the starting point (pre D) are summarized in Figure 3.

**Figure 4:**
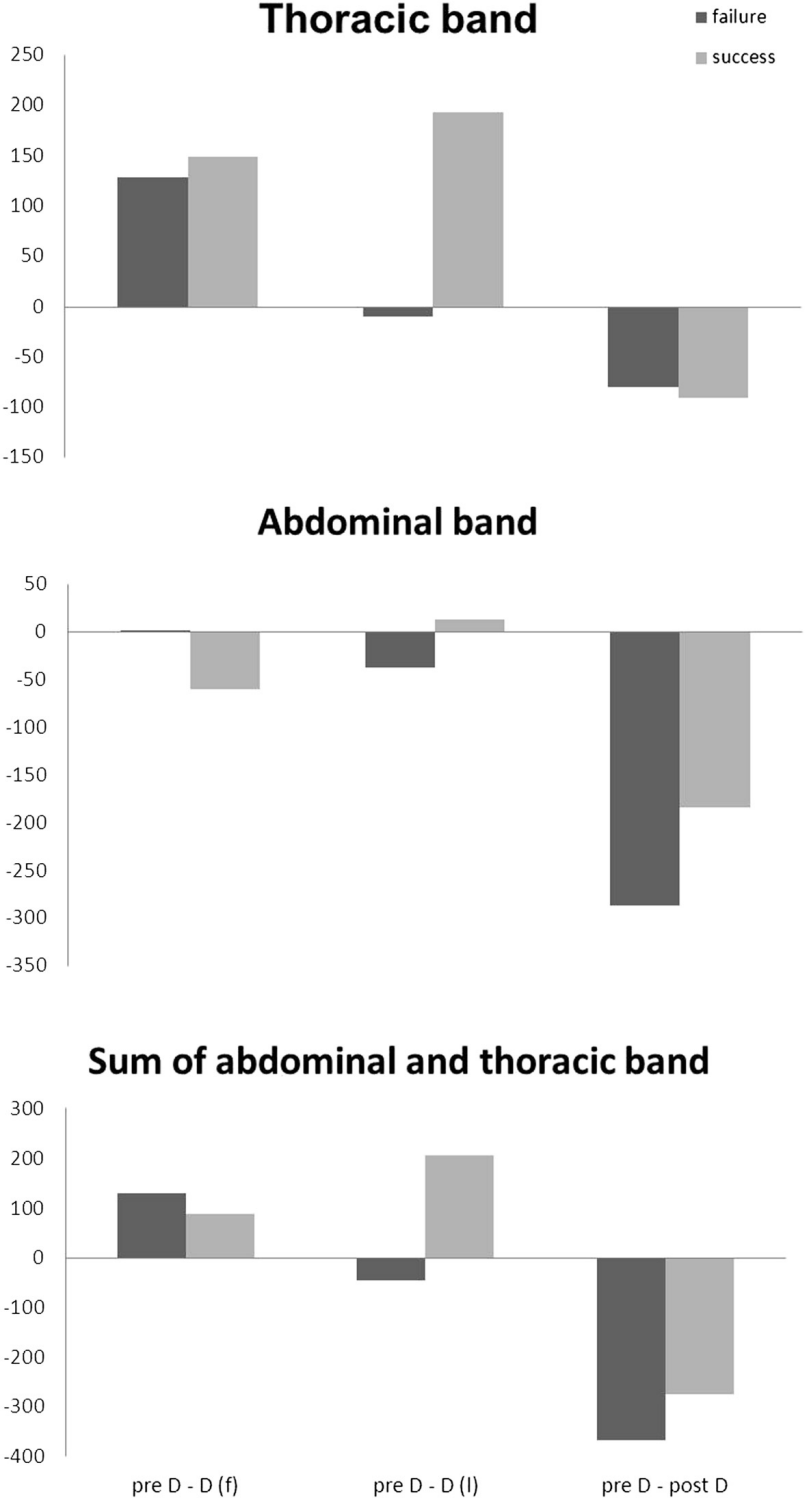
**Respiratory inductive plethysmography (RIP) data illustrating changes in lung volume in relation to pre D during the spontaneous breathing trial (in arbitrary units).** The figure shows RIP data during an SBT, presented as differences from pre-disconnetion values. Therefore, positive differences stand for a decrease in RIP, and negative differences stand for an increase in RIP. Dark columns represent failure patients, and light columns, the success patients. Please note: Compared with preD, there is a decrease in RIP values to D(f), and D(l); only the failure patients show increased RIP values.

The analysis of the RIP data consisted of the thoracic (RIP_TX_) and abdominal band (RIP_AB_) and the sum of the two (RIP_SUM_) (Figure 4). Adding the changes of the thoracic and the abdominal band can be used to illustrate overall changes in functional residual capacity (FRC). This summation value decreases after disconnecting the patients from the ventilator (D(f)), reflecting a decrease in thoracic circumference. In the failure patients, an increase in the summation of both bands toward the end of the SBT (D(l)) is visible. After reconnection to the ventilator, this value reflects an increase compared with the pre-disconnection level in all patients, which is more pronounced in the failure patients.

## Discussion

Our observations show that EAdi can be used as additional monitoring when weaning difficult-to-wean patients from the ventilator.

During the SBT, we assessed the patients by using a standard protocol [18]. The patient was thereby completely disconnected from the ventilator to see inspiratory muscle activity during the whole inspiratory period and to avoid effects of triggering pressure support by only brief diaphragmatic contractions [24]. Conventional criteria were used to decide when to terminate the SBT. Six of the patients failed the SBT, and 12 passed. The EAdi differed between the two groups, with an early increase in the failure patients that was more pronounced than that in those who passed the SBT. Our findings confirm those of Dres and colleagues [15], who described an early increase of EAdi-derived indices (such as EAdi_max_/VT) in patients who failed a weaning trial. The result is reflected by the decreasing VT/EAdi ratio in the failure group. In an animal model, Grasselli and co-workers [5] demonstrated that the (VT/EAdi, no-assist)/(VT/EAdi, assist) ratio decreases with increasing ventilatory support, as does the work of breathing. Although evaluation of EAdi confirms physiological concepts that an increase of EAdi can be expected in a failing diaphragm, it does not outperform respiratory rate-derived indices in our study. The potential benefits of EAdi might be visible when the parameter is assessed continuously, helping to detect pathophysiologic changes early. Figure 1 shows the anticipated increase of EAdi in a failure patient.

A recent study by Wolf and co-workers [16] used the ratio VT/(EAdi_max __ EAdi_min_) to assess extubation readiness in a pediatric population. A lower ratio of tidal volume to deltaEAdi was found in the patients who passed the SBT, suggesting that patients with a higher deltaEAdi relative to tidal volume at the end of a 1-hour SBT may have better-preserved diaphragmatic function and therefore may be more easily weaned from ventilator support. Our observations showed that the patients who failed the SBT had an early reduction in the VT/EAdi ratio that was mainly because of a steep increase in EAdi. This could be explained by the patients’ efforts to maintain sufficient tidal ventilation.

Provided intact feedback exists to the patients’ central respiratory regulation, patients experience a decrease in minute ventilation (via increasing PaCO_2_) and lung volumes (for example, FRC (via PaO_2_ and stretch receptors)) when disconnected from the ventilator. Those who are unable to generate a sufficient tidal volume and maintain the FRC will increase inspiratory drive and thus neural output to the diaphragm. Insufficient increase in muscle effort will induce a further increase in EAdi, with ultimate failure of the SBT. The VT/EAdi ratio may be affected by increased resistive load (for example, upper airway obstruction), decreased pulmonary or chest-wall compliance (for example, pulmonary edema, obesity), mechanical alterations of diaphragmatic function (for example, ventilator-induced diaphragmatic dysfunction [25]) and reduced neuromuscular coupling (for example, critical illness neuromuscular abnormalities [17,26,27]). Under such circumstances, each patient has an individual threshold of respiratory efficacy, reflected by the VT/EAdi ratio, corresponding to the idea of a “fatigue zone” from the work of Tobin [28].

The study of Wolf *et al.*[16] differed from ours with regard to patient population and study design (1-hour SBT with PEEP reduced to 5 cm H_2_O and pressure support reduced to overcome resistance of endotracheal tube), but we agree that the VT/EAdi ratio reflects the effectiveness of diaphragmatic activation and may be a useful parameter in predicting successful weaning, although the results must be interpreted with caution. The parallel evaluation of a pressure-derived weaning index (for example, *P* = 0.1) may elucidate underlying reasons for weaning failure.

We compared the EAdi and VT/EAdi with the RR/VT ratio, which is the most commonly used and best-established predictor of weaning [29]. The RR/VT ratio is influenced by PSV and PEEP [30,31]. Therefore, we measured the RR/VT ratio during a T-piece trial, to maintain its typical cut-off value, although no difference appeared in the outcome of SBT either PSV or a T-piece [32,33].

The failure and success groups differed greatly with regard to the two ratios, RR/VT and VT/EAdi, but the differences were not statistically significant. This could be attributable to the small population size. The failure group reached a median value of >105 bpm/L after disconnection form the ventilator, indicating extubation failure. However, because the RSBI was not available at the bedside during the trial, the decision to terminate the SBT was based on the clinical criteria described earlier. The decision to discontinue was reached relatively late (18 (8/22) minutes) compared with the early increase in EAdi at D (f) after 5 minutes.

The results of the RIP data add to the recent study by Dres and colleagues [23] on this matter and indicate that lung volume decreases the patient is disconnected from the ventilator (Figure 4, pre D-D(f)); mainly as a result of decreased thoracic circumference. This may be interpreted as because of loss of PEEP and pressure support during disconnection [23]. Lung volume increased toward the end of the SBT in the patients who failed the SBT (Figure 4, preD-D(l)). A possible explanation is the rapid, shallow breathing that these patients increasingly exhibit. Combined with expiratory-flow limitation, which has been demonstrated in patients with respiratory failure [34], this is the leading mechanism responsible for the development of intrinsic PEEP [35]. The additional effort is also indicated by the increase in EAdi. When the patient was reconnected to the ventilator, the thoracic and abdominal circumferences increased in the patients in both groups, probably because of the applied PEEP. The effect is more pronounced in the failure patients, which may indicate intrinsic PEEP.

### Study limitations

This study has several limitations. It was planned as an observational pilot trial and was not powered to give significant results. The observed large differences in some parameters are difficult to interpret because of the small population size. We excluded patients who were considered simple to wean [17]. All of our patients were in the process of being weaned from the ventilator. Ten patients had had at least one unsuccessful extubation attempt and had been given a tracheotomy. The heterogeneity of the collective and limiting our study to difficult-to-wean patients makes a generalization of our results difficult, but it underlines the feasibility and informative value of EAdi as a monitoring parameter. Most of our patients had been admitted to the ICU after cardiothoracic surgery, a factor that may have contributed to the underlying cause of weaning failure.

The VT data and derived indices during disconnection from the ventilator must be used cautiously, as the tidal volume was estimated from the RIP data in the first 11 patients. For calibration, we used the QDC described by Sackner [21]. It uses the equation: dVT = M(K × dRIP_TX_ + dRIP_AB_ (dVT, delta tidal volume; M, constant factor, scaling the summation of RIP_TX_ and RIP_AC_ to volume; K, calibration factor, indicating the relative contribution of RIP_TX_ and RIP_AB_ to volume; dRIP_TX/AB_, = delta of the respiratory inductive plethysmography reading of the thorax/abdomen). Although this has been validated in naturally breathing subjects [21,36], transferring the method to critically ill patients being disconnected from a ventilator must be regarded as an approximation and may have underestimated the tidal volume during disconnection. With a fixed weighting of the RIP signals (K = 1), measurement errors must be taken into ac-count [37].

## Conclusion

Continuous EAdi monitoring provides additional information for the clinician caring for patients during weaning. We found that EAdi shows an early increase during the SBT in patients who failed the trial, and this increase can be detected before a patient finally fails according to clinical criteria. Thus, EAdi monitoring has the potential to guide respiratory weaning in difficult-to-wean patients and might help to prevent the muscular exhaustion during weaning trials, known to have detrimental effects on the patient. Further studies of the EAdi signal during liberation from mechanical ventilation and comparison with other weaning parameters is needed.

## Key messages

• EAdi monitoring provides valuable information on respiratory drive.

• During weaning from the ventilator, EAdi monitoring may help to identify patients who are not ready for discontinuation of respiratory support.

• EAdi monitoring might help to prevent the muscular exhaustion during weaning trials that is known to have detrimental effects on the patient.

• Further analysis of the EAdi and comparison with other weaning parameters is needed.

## Abbreviations

BMI: Body mass index; BP: Systolic blood pressure; bpm: Beats per minute; D(f): 5 minutes after the patient was disconnected (first measurement during SBT); D(l): Before reconnecting the patient to the ventilator (last measurement during SBT); EAdi: Electrical activity of the diaphragm; FRC: Functional residual capacity; HR: Heart rate; ICU: Intensive care unit; MAAS: Motor Activity Assessment Scale; NAVA: Neurally adjusted ventilatory assist; PEEP: Positive end-expiratory pressure; post D: 5 minutes after reconnecting the patient to the ventilator; pre D: 5 minutes before the patient was disconnected from the ventilator; PSV: Pressure-support ventilation; QDC: Qualitative diagnostic calibration; RIP: Respiratory-inductive plethysmography; RIPAB: Respiratory-inductive plethysmography data from the abdominal band; RIPSUM: Respiratory-inductive plethysmography data from the summation of both bands; RIPTX: Respiratory-inductive plethysmography data from the thoracic band; RR: Respiratory rate; RSBI: Rapid shallow breathing index; SAPS II: New Simplified Acute Physiology Score; SBT: Spontaneous breathing trial; VT: Tidal volume; VTest: Tidal volume estimated by respiratory-inductive plethysmography (patients 1 to 11).

## Competing interests

The Department of Anesthesiology, Emergency and Intensive Care Medicine, University of Göttingen, provides educational courses on lung-protective ventilation that are supported in part by Maquet Critical Care. In 2006, Dr. Moerer participated in a research project at Uppsala University, partly financed by research grants of Göttingen and Uppsala University and Maquet Critical Care. Prof. Quintel consults for companies that manufacture ventilators, including Maquet Critical Care, and is remunerated for these consultations.

## Authors’ contributions

JB designed the study, participated in the bedside measurements, coordinated the offline analysis, performed the statistical analysis, and wrote the manuscript. CP and UO performed the bedside measurements and participated in the data analysis and drafting the manuscript. MQ participated in the design of the study. OM conceived the study and participated in its design, participated in the bedside measurements and in writing the manuscript, and coordinated the study. All authors read and approved the final manuscript.
